# Supplementary material for: Comparing methods for creating a national random sample of twitter users
Source: Soc Netw Anal Min. 2024 Aug 14;14(1):160. doi: 10.1007/s13278-024-01327-5 (PMC11861139; doi:10.1007/s13278-024-01327-5)
Supplement: Supplementary file 1 — Supplementary file1 (pdf 807 kb) [file 13278_2024_1327_MOESM1_ESM.pdf]

Random User ID Generation Sampling Method

Twitter has two distinct types of IDs - the older 32-bit serial IDs and the newer 64-bit random IDs. To ensure the validity of the sample, we developed a function that is capable of generating both types of IDs.

To gather a representative sample of user IDs, we use an iteration function that generates both old 32-bit IDs and new 64-bit IDs. For each iteration, we set a random seed generated from a seed generator. This ensures reproducibility if the process is rerun on any system. During each iteration, we generate 1000 IDs, with 5 being old IDs and 995 being new IDs. The distribution is heavily skewed towards new IDs due to their higher hit rate compared to old IDs. The old IDs are simply random 32-bit integers that have not been generated before. For 64-bit IDs, we employ the snowflake approach, which generates unique IDs and is also used by Twitter [6]. This approach generates valid user IDs while avoiding reserved IDs for future use. Twitter created the Twitter Snowflake ID generator to produce unique IDs for objects needing identification, such as tweets and users. Twitter Snowflake optimizes the traditional UUID concept to work on a large scale while maintaining sortable IDs and manageable sizes. Twitter Snowflake IDs are 64-bit long and sortable by time, making them ideal for indexing and efficient in storage and processing. The system is scalable, accommodating up to 1024 machines, and highly available, with each machine capable of generating 4096 unique IDs per millisecond. The fact that the IDs are time-dependent makes it possible to generate working IDs with a higher chance since one can restrict the generation to only IDs from the past (Pointer).

We Found 357K users with Random ID and filtered the American users with query *place country:US*. However, we got only 127 users from the US. Finding working IDs from a specific country with a country code is tricky. Thus, getting a large enough sample from random user IDs is almost impossible due to the low number of calls one can make to the API and the fact that with the random ID assignment, the number of invalid ID strings is tiny compared to the actual number of possible IDs. In fact, there are 1024 possible IDs for each millisecond, and on average, between zero to two accounts are valid (we found it from testing). Hence, a random user ID seldom returns a match. Therefore, we did not include this method in this paper.

[6] https://developer.twitter.com/ja/docs/basics/twitter-ids

Robustness Tests

Regression Settings: Including District of Columbia

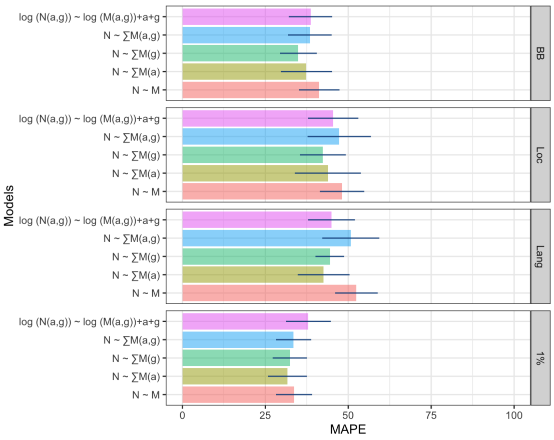

Figure S1: Performance on leave one state out population inference across different debiasing models, where the District of Columbia is included and rows with all zero value were removed from the regression. The bar shows MAPE(N) robust standard errors clustered on states.

Regression Settings: Removing All States With At Least One Missing Group

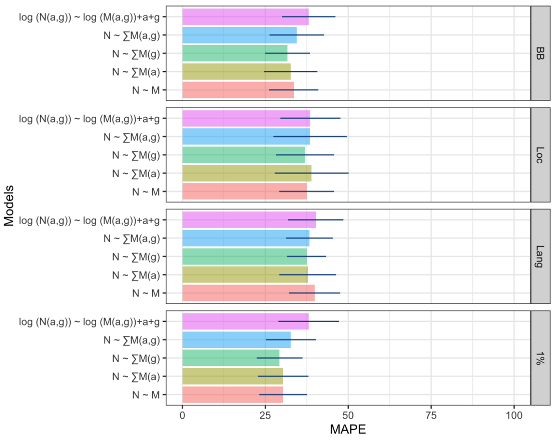

Figure S2: Performance on leave one state out population inference across different debiasing models, where all states with at least one missing demographic group in their Twitter data is removed from the regression. The bar shows MAPE(N) robust standard errors clustered on states.

Regression Settings: Leave-One-Division-Out

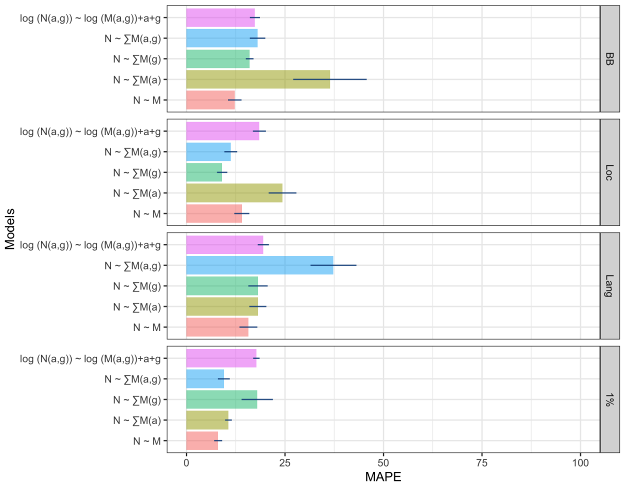

Figure S3: Performance on leave one division out population inference across different debiasing models. The bar shows MAPE(N) robust standard errors clustered on US divisions.

Pre-Processing Settings: Removing Users with Less than 200 Tweets

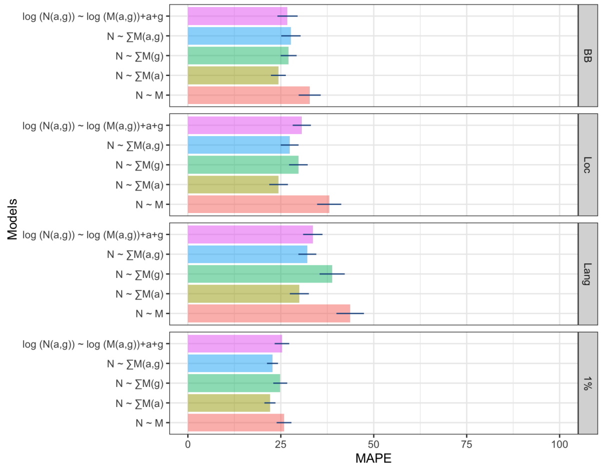

Figure S4: Performance on leave one state out population inference across different debiasing models where rows with all zero value were removed from the regression and minimum number of tweets filter changed from 100 to 200. The bar shows MAPE(N) robust standard errors clustered on states.

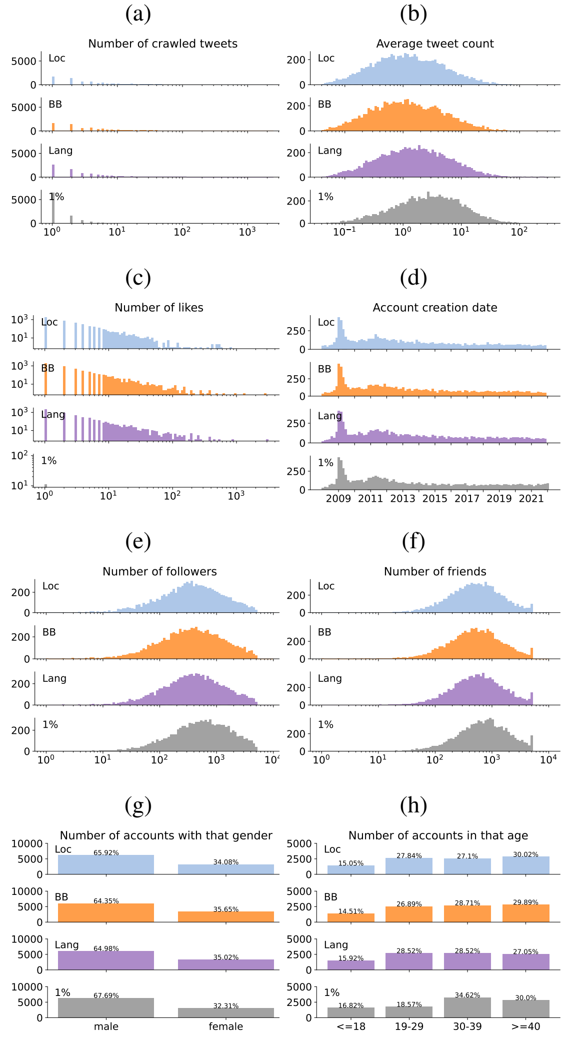

Figure S5: Distributions of (A) number of tweets; (B) average number of tweets per day; (C) number of likes; (D) account creation date; (E) number of followers; and (F) number of friends for different groups. Distribution of users with respect to (G) gender and (H) age across the four Twitter sampling methods after changing the minimum number of tweets filter from 100 to 200. After removing accounts with more than 200 tweets, the sample size decreased to 9,437. Consequently, we randomly selected an equivalent sample size for the remaining three samples.

Pre-Processing Settings: Removing Users with Less than 1 Year Account Age

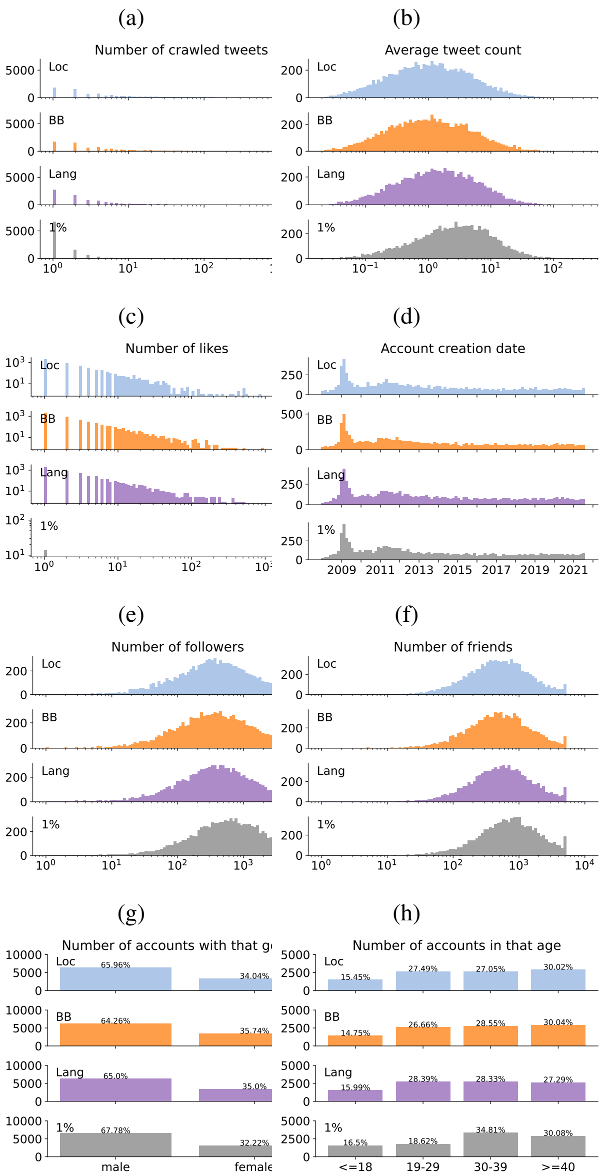

Figure S6: Distributions of (A) number of tweets; (B) average number of tweets per day; (C) number of likes; (D) account creation date; (E) number of followers; and (F) number of friends for different groups. Distribution of users with respect to (G) gender and (H) age across the four Twitter sampling methods after changing the minimum age of accounts filter from 9 months to one year. After excluding accounts created within the past year, the sample size was further reduced to 9,691. Subsequently, we matched this sample size for the remaining three samples.

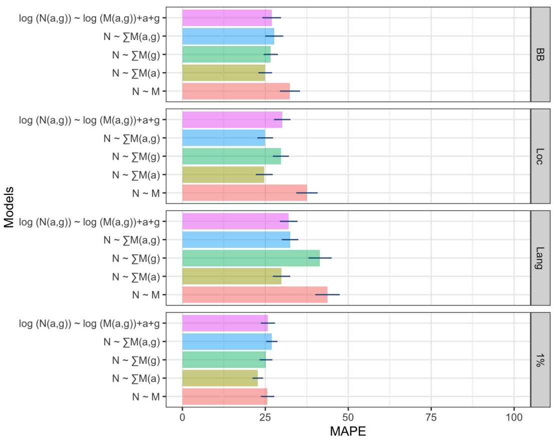

Figure S7: Performance on leave one state out population inference across different debiasing models where rows with all zero value were removed from the regression and the minimum age of accounts filter changed from 9 months to one year. The bar shows MAPE(N) robust standard errors clustered on states.
